# Blood neuroexosomal excitatory amino acid transporter-2 is associated with cognitive decline in Parkinson’s disease with RBD

**DOI:** 10.3389/fnagi.2022.952368

**Published:** 2022-08-23

**Authors:** Bing Leng, Hairong Sun, Mengfan Li, Junwu Zhao, Xiaoxiao Liu, Ran Yao, Tengqun Shen, Zhenguang Li, Jinbiao Zhang

**Affiliations:** Department of Neurology, Weihai Municipal Hospital, Cheeloo College of Medicine, Shandong University, Weihai, China

**Keywords:** Parkinson’s disease, REM sleep behavior disorder, cognitive dysfunction, vesicular glutamate transporter-1, excitatory amino acid transporter-2

## Abstract

**Background:**

Rapid eye movement (REM) sleep behavior disorder (RBD) predicts cognitive decline in Parkinson’s disease (PD) patients without dementia. However, underlying mechanisms remain unknown. Accumulating studies suggest glutamatergic system dysregulation is associated.

**Objective:**

To examine the effect of RBD on the rate of cognitive decline in PD patients and investigate whether plasma levels of the neuroexosomal vesicular glutamate transporter-1 (VGLUT-1) and excitatory amino acid transporter-2 (EAAT-2) are altered in PD patients with RBD.

**Methods:**

This study included 157 newly diagnosed cognitive normal PD patients and 70 healthy controls (HCs). Based on one-night polysomnography recordings, the PD subjects were divided into PD with and without RBD (PD-RBD and PD-nRBD) groups. All participants received a complete clinical and neuropsychological evaluation at baseline. Plasma levels of neuroexosomal VGLUT-1 and EAAT-2 were measured by ELISA kits. After a 3-year follow-up, we evaluated baseline plasma levels of neuroexosomal glutamate transporters in each group as a predictor of cognitive decline using MoCA score changes over 3 years in regression models.

**Results:**

Plasma levels of neuron-derived exosomal EAAT-2 and VGLUT-1 were significantly lower in PD patients than in HCs. Plasma levels of neuroexosomal EAAT-2 were significantly lower in PD-RBD than PD-nRBD group at baseline. At the 3-year follow-up, PD-RBD patients presented greater cognitive decline. Lower baseline blood neuroexosomal EAAT-2 predicted cognitive decline over 3 years in PD-RBD patients (β = 0.064, *P* = 0.003).

**Conclusion:**

These findings indicate that blood neuroexosomal EAAT-2 is associated with cognitive decline in PD with RBD.

## Introduction

Cognitive impairment (CI) is one of the most common and important non-motor symptoms in Parkinson’s disease (PD), occurring in as many as 80% of patients in the long term (Hely et al., 2008), and is associated with poor outcomes (de Lau et al., 2005). During the past decade, there has been increased focus on the predementia stages of CI in PD patients. Early identification of individuals at risk of developing CI could help stratify the PD population for prognostic information and treatment selection and improve understanding of the pathophysiology of cognitive decline in these patients. Rapid eye movement (REM) sleep behavior disorder (RBD) is found in up to 60% of patients with PD and has been identified as a clinical risk factor for cognitive decline and dementia in PD. Early studies have shown that RBD predicts cognitive decline in early-stage and advanced PD without dementia (Vendette et al., 2007; Chahine et al., 2016; Liu et al., 2019). However, the underlying mechanism for the association remains unknown. Accumulating clinical and experimental data suggest that RBD might be due to the neurodegeneration of glutamate neurons involved in paradoxical sleep (Luppi et al., 2011, 2013; Wang et al., 2021). The process of human memory consolidation is strongly dependent on REM sleep. In addition, the activity of glutamate ionotropic receptors is known to mediate both synaptic plasticity and the consolidation of various types of memories. The glutamatergic system may be associated with cognitive decline in PD with RBD.

Hyperactivity of the glutamatergic system plays a role in the pathophysiology of PD. Nigrostriatal dopaminergic depletion causes overactivity of the glutamatergic projections to the basal ganglia output nuclei from the corticostriatal pathway and the subthalamic nucleus and increases striatal release of glutamate (Buchanan et al**.**, 2015; Iovino et al., 2020). A study using magnetic resonance spectroscopy showed dysregulation of glutamatergic neurotransmission in several brain regions in patients with PD (Weingarten et al., 2015). Glutamate is transported into synaptic vesicles by vesicular glutamate transporters, released at the synaptic cleft and taken up via specific glutamate transporters, thus terminating glutamate function at the synapse. It was reported in mice that following progressive 1-methyl-4-phenyl-1,2,3,6-tetrahydropyridine (MPTP) treatment, glutamate transporters, including vesicular glutamate transporter-1 (VGLUT-1) and glutamate transporter-1 (GLT-1) (named excitatory amino acid transporter-2, EAAT-2, in the human brain), were significantly increased (Sconce et al., 2015). A recent study reported that anti-N-methyl-D-aspartate receptor (NMDAR) encephalitis is associated with parkinsonism-like symptoms, RBD and CI (Çoban et al., 2014). According to these findings, a plausible hypothesis is that glutamatergic hyperactivation may exert a crucial effect on cognitive dysfunction in PD patients with RBD.

Exosomes are nanosized extracellular vesicles that can be released into the extracellular matrix by most cell types and can be found in many biofluids, such as blood, CSF, saliva, and urine. Their encapsulated cargos can reflect the intracellular environment of the originating cells and participate in intracellular communication under various physiological and pathological conditions (van Niel et al., 2018). Exosomes can cross the blood–brain barrier and can be detected in the plasma (van Niel et al., 2018). The recent development of methods for the isolation of neuron-derived exosomes (NDEs) from plasma has permitted the quantification of neuronal proteins relevant to the pathogenesis of human neurodegenerative diseases (Mustapic et al., 2017).

In this study, we aimed to examine the effect of RBD on the rate of cognitive decline in PD patients and investigate whether plasma levels of the neuroexosomal glutamate transporters VGLUT-1 and EAAT-2 are altered in PD patients with RBD.

## Materials and methods

### Participants

The diagnosis of PD was based on the clinical diagnostic criteria for Parkinson’s disease from 2015 formulated by the Movement Disorder Society in the United States (Postuma et al., 2015). The inclusion criteria were as follows: patients with idiopathic PD diagnosed by experienced neurologists according to these diagnostic criteria; patients who were previously untreated; and patients who were cognitively normal [determined by a Montreal Cognitive Assessment (MoCA) score ≥ 26] (Hoops et al., 2009).

The exclusion criteria were as follows: patients with atypical features that indicate progressive supranuclear palsy, multiple system atrophy, corticobasal degeneration, cerebellar signs, supranuclear gaze palsy, apraxia, and disabling autonomic failure; patients with parkinsonian syndrome secondary to stroke, trauma, or other neurological and psychiatric disorders; patients with malignant tumors, disabilities, or other severe physical diseases; patients who underwent surgical treatment, deep brain stimulation, stem cell transplantations, or other medical treatment; patients with other causes of CI (such as delirium, stroke, severe depression, metabolic disorders, drug side effects, and head trauma); patients with PD comorbidities such as severe movement disorders, severe anxiety [Hamilton Anxiety Scale (HAMA) score ≥ 21] (Hamilton, 1959), severe depression [Hamilton Depression Scale 17 items (HAMD-17) score ≥ 24] (Zimmerman et al., 2013), excessive daytime sleepiness, and psychiatric disorders that may have effects on the cognition assessment.

**FIGURE 1:**
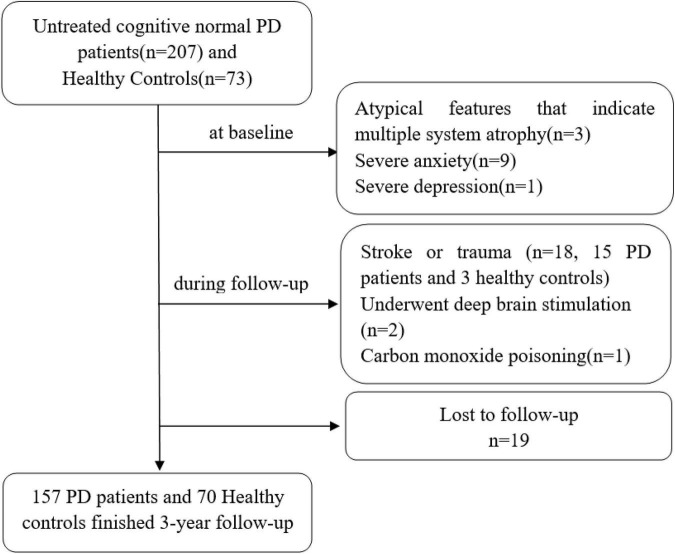
Description of the study population. PD indicates Parkinson’s disease.

According to the above inclusion criteria, 207 PD patients were enrolled in our study from May 2016, to September 2018. Thirty-one of them were excluded at the start of the study and during the follow-up according to the exclusion criteria, 19 were lost to follow-up, and 157 completed 3 years of follow-up neuropsychological assessments every 6 months (shown in Figure 1).

Healthy controls (HCs) were recruited from the spouses and friends of the PD participants and were matched for age and sex. The control individuals had no cognitive impairment, no psychiatric disorders, such as anxiety and depression, no history of severe physical disorders, normal thyroid gland function and renal function, no physical disabilities, and no hearing or visual impairment. Seventy HCs completed the 3-year follow-up.

All the subjects underwent clinical and neuropsychological assessments, polysomnography (PSG) recordings and blood collection at baseline.

The study was conducted with approval from the Ethics Committee of Weihai Municipal Hospital. Written informed consent was obtained from all participants.

### Clinical and neuropsychological assessments

Motor disability was staged using the Hoehn and Yahr (H&Y) scale. Motor symptoms were elevated using the Movement Disorder Society Unified Parkinson’s Disease Rating Scale Part III (MDS-UPDRS-III). Psychological status was assessed by the HAMA and HAMD-17. Cognitive status was assessed with the Beijing version of the MoCA. The range of total scores on this scale is 0–30 points. If the subject had received 12 years of education or less, 1 point was added to correct for the bias of the educational level. The recommended cutoff score of < 26 was used to define CI (Nasreddine et al., 2005). A score ≥ 26 points was considered cognitively normal. The Δ% MoCA over 3 years was defined as $\Delta \%\,\mathrm{MoCA}=\frac{\text{MoCAfollow}-\text{up}-\mathrm{MoCAbaseline}}{\text{MoCAbaseline}}\times 100\%$ and used to reflect cognitive decline in each group. In addition, individual subdomains of MoCA tests were also recorded. All clinical and neuropsychological assessments were conducted between 7:00 and 10:00 in the morning, and the patients stopped taking their medication the night before the follow-up evaluation. Levodopa equivalent dosages (LEDs) were calculated using a standard formula (Tomlinson et al., 2010). All of the evaluation tests were conducted by two experienced neurologists who did not have knowledge of the grouping situation of the patients.

### RBD sleep assessments

All participants underwent one-night PSG recordings in the sleep laboratory. Sleep was recorded using a polygraph composed of eight EEG electrodes: two frontal (F3/A2, F4/A1), two central (C3/A2, C4/A1), two temporal (T3/A2, T4/A1) and two occipital (O1/A2, O2/A1) electrodes. Left and right electrooculograms were used to measure eye movements, and submental electromyography was used to measure muscle activity. Oral and nasal airflow and thoracic and abdominal movements were recorded, and oximetry was performed to exclude sleep apnea and hypopnea syndrome. RBD was diagnosed by a sleep specialist according to the criteria of the International Classification of Sleep Disorders, Second Edition and PSG criteria (Montplaisir et al., 2010).

### Blood sample preparation and neuron-derived exosomes isolation

Two milliliters of venous blood was drawn from each participant into a polypropylene tube with EDTA in the morning after a 10-h fast at base line. The samples were centrifuged for 15 min at 3,000 × *g* to obtain plasma. NDEs were immediately separated according to a published protocol (Goetzl et al., 2016). In brief, aliquots of 0.5 ml plasma were incubated with 0.15 ml thromboplastin D (Thermo Fisher Scientific, Waltham, MA, United States) for 60 min, followed by the addition of 0.25 ml of calcium- and magnesium-free Dulbecco balanced salt solution with protease and phosphatase inhibitor cocktail (Thermo Fisher Scientific). The mixed solution was centrifuged at 1,500 *g* for 30 min at 4°C. The supernatants were then mixed with ExoQuick exosome precipitation solution (EXOQ; System Biosciences, CA, United States) and incubated for 1 h on ice. After centrifugation at 3,000 *g* for 30 min at 4°C, each pellet was resuspended in 250 μl DPBS. Each sample was mixed with 100 μl 3% bovine serum albumin (BSA, Thermo Fisher Scientific) and incubated for 1 h on ice with 3 μg mouse anti-human CD171 (L1CAM neural adhesion protein) biotinylated antibody (clone 5G3; eBioscience, San Diego, CA, United States), followed by incubation with 25 μl Streptavidin-Plus UltraLink resin (Thermo Fisher Scientific) in 50 μl of 3% BSA. After centrifugation at 400 *g* for 10 min and removal of supernatant, each pellet was resuspended in 50 μl of 0.05 M glycine-HCl (pH 3.0), incubated at 4°C for 10 min, and centrifuged at 4°C for 10 min at 4,000 *g*. Supernatants were transferred to prechilled Eppendorf tubes that contained 10 μl of 1 M Tris–HCl (pH 8.0) and 25 μl of 10% BSA, mixed before addition of 365 μl M-PER mammalian protein extraction reagent (Thermo Fisher Scientific) that contained protease and phosphatase inhibitors, and stored at −80°C before enzyme-linked immunosorbent assay (ELISA) analysis.

**FIGURE 2:**
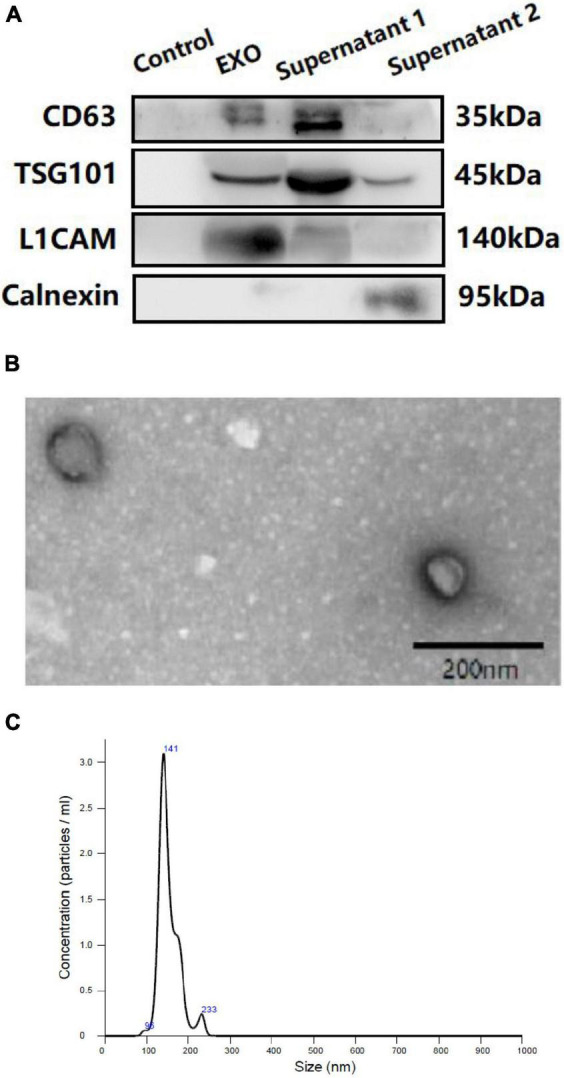
Western blotting (WB) **(A)**, transmission electron microscopy (TEM) **(B)** and nanoparticle tracking analysis (NTA) **(C)** of plasma neuron derived exosomes. **(A)**: Control: negative control for the immunoprecipitation using Streptavidin-Plus UltraLink resin alone without mouse anti-human CD171 biotinylated antibody. EXO: neuron-derived exosomes. Supernatant 1: Supernatant after immunoprecipitation. Supernatant 2: Supernatant after ExoQuick exosome precipitation.

Western blotting (WB), transmission electron microscopy (TEM), and nanoparticle tracking analysis (NTA) were performed to confirm the success of exosomal collection (shown in Figure 2) in accordance with our previous protocols (Zhang et al., 2021; Chi et al., 2022).

### Determination of plasma neuron-derived exosomal vesicular glutamate transporter-1 and excitatory amino acid transporter-2 levels

The NDE proteins were quantified by ELISA kits for the exosome markers CD63 (ELH-CD63-1, Ray Biotech Inc., Norcross, GA, United States), VGLUT-1 (abx553000; Abbexa, United Kingdom) and EAAT-2 (abx151369; Abbexa, United Kingdom). The mean value for all the measurements of CD63 was set at 1.00, and the relative values of CD63 for each sample were used to normalize the total NDE-protein contents. One investigator conducted all ELISAs without knowledge of the clinical data for any subject.

### Statistical analysis

Parkinson’s disease patients were divided into PD with and without RBD groups (PD-RBD and PD-nRBD). Baseline demographic and MoCA scores and other demographic and clinical data were compared between PD-RBD and PD-nRBD participants using two-tailed independent *t*-test for continuous variables and chi-square (χ^2^) tests for categorical variables. Univariate analyses of variance were applied to compare demographic, clinical, and mood variables among PD-RBD, PD-nRBD, and healthy controls. Independent *t*-tests were applied to compare plasma levels of NDE EAAT-2 and VGLUT-1 between each group. Normality assumptions were checked where appropriate. Kaplan-Meier survival analysis was used to estimate the effects of RBD on the progression to CI. Δ% MoCA was compared between each group using independent *t*-test.

We analyzed the associations between all baseline plasma biomarkers and Δ% MoCA in each group. Linear regression models evaluated the predictive effects of all baseline plasma biomarkers on outcomes of Δ% MoCA over 3 years in each group. Confounding variables based on biological rationality and published data were selected in a linear regression model. Because UPDRS part III scores differed between the PD RBD and non-RBD subjects, a linear regression model was corrected for UPDRS part III scores when analyzing the association between all baseline plasma biomarkers and Δ% MoCA. Age and education were also included as covariates for the analyses involving cognition. Statistical analysis was performed using SPSS (version 21.0), and a *P* value < 0.05 was considered statistically significant.

## Results

### Baseline demographic and clinical characteristics of the study subjects

The baseline demographic and clinical characteristics of all the participants are presented in Table 1. MoCA scores at baseline are shown in Table 2. Among the 157 PD patients, 91 (57.96%) had PD without RBD, and 66 (42.04%) had PD with RBD. There were no differences in age, sex, education, MoCA scores, HAMA scores or HAMD scores among the three groups. Between the PD-RBD and PD-nRBD groups, there were no differences in disease duration or Hoehn and Yahr stage, but there was a significant difference in the UPDRS part III scores between the groups. The PD-RBD group had higher UPDRS part III scores. Each subdomain of MoCA scores was also analyzed. At baseline, PD-RBD patients performed worse than PD-NRD patients and HCs in tests of visuospatial function (*P* = 0.022) and memory (*P* = 0.044).

**TABLE 1:** Baseline demographic and clinical characteristics of PD patients stratified by RBD and Healthy controls.

	PD-RBD (*n* = 66)	PD-nRBD (*n* = 91)	HCs (*n* = 70)	*P*-value
Age (years)	65.95 ± 5.45	65.14 ± 5.69	62.38 ± 9.54	0.279
Sex (male, n%)	43.64.2%	58.64%	44.63.4%	0.531
Education (years)	9.38 ± 2.98	9.25 ± 2.99	9.89 ± 3.13	0.157
Disease duration (months)	16.97 ± 10.14	16.10 ± 7.43	–	0.535
Hoehn and Yahr Stage			–	0.334
Stage 1	21, 31.8%	28, 30.8%	–	
Stage 1.5	7, 10.6%	10, 11.0%	–	
Stage 2	17, 25.8%	34, 37.4%	–	
Stage 2.5	21, 31.8%	19, 20.9%	–	
UPDRS part III	25.32 ± 9.22	22.13 ± 9.68	–	0.034*
LED (mg/d)	401 ± 205	375 ± 199	–	0.451
HAMA	19.25 ± 2.99	18 ± 5.92	17.43 ± 3.47	0.574
HAMD	11.50 ± 4.66	8.86 ± 5.61	8.79 ± 3.44	0.428

Results are mean ± (SD). Univariate analyses of variance was applied to compare demographic, clinical, and mood variables among PD-RBD, PD-nRBD, and healthy controls. Independent t-test was used to compare disease duration and Levodopa equivalent dosages between PD-RBD and PD-nRBD group. Gender and Hoehn and Yahr Stage between each group were compared using chi-square (χ^2^) test. *P value statistically significant.

**TABLE 2:** MoCA scores at baseline and at the end of the 3-year follow-up.

	PD-RBD (*n* = 66)	PD-nRBD (*n* = 91)	HCs (*n* = 70)	*P*-value
MoCA-baseline	28.08 ± 1.40	28.30 ± 1.15	27.69 ± 1.52	0.153
Visuospatial function-baseline	3.77 ± 0.58	3.97 ± 0.57	4.64 ± 0.49	0.022*
Naming-baseline	2.95 ± 0.21	2.98 ± 0.15	2.97 ± 0.16	0.569
Memory-baseline	4.24 ± 0.70	4.45 ± 0.56	4.34 ± 0.84	0.044*
Attention & Calculation-baseline	5.70 ± 0.46	5.59 ± 0.51	5.69 ± 0.52	0.243
Language-baseline	2.86 ± 0.35	2.91 ± 0.29	2.75 ± 0.44	0.135
Abstraction-baseline	1.69 ± 0.46	1.74 ± 0.44	1.76 ± 0.48	0.478
Orientation-baseline	6.00 ± 0.00	6.00 ± 0.00	5.99 ± 0.08	0.897
MoCA-3-year	24.41 ± 2.44	25.43 ± 2.38	25.94 ± 1.62	0.009*
Visuospatial function-3-year	3.21 ± 0.69	3.58 ± 0.73	4.43 ± 0.55	0.003*
Naming-3-year	2.90 ± 0.77	2.93 ± 0.21	2.97 ± 0.25	0.749
Memory-3-year	3.10 ± 0.94	3.20 ± 0.93	3.23 ± 0.70	0.477
Attention & Calculation-3-year	5.26 ± 0.71	5.46 ± 0.62	5.79 ± 0.46	0.019*
Language-3-year	2.38 ± 0.54	2.60 ± 0.52	2.61 ± 0.51	0.058
Abstraction-3-year	1.49 ± 0.64	1.61 ± 0.53	1.69 ± 0.56	0.199
Orietation-3-year	5.95 ± 0.21	5.98 ± 0.11	5.97 ± 0.16	0.395

Results are mean ± (SD). Univariate analyses of variance was applied to compare MoCA scores and subdomains among PD-RBD, PD-nRBD, and healthy controls. *P value statistically significant.

### Baseline glutamate-related transporters

**FIGURE 3:**
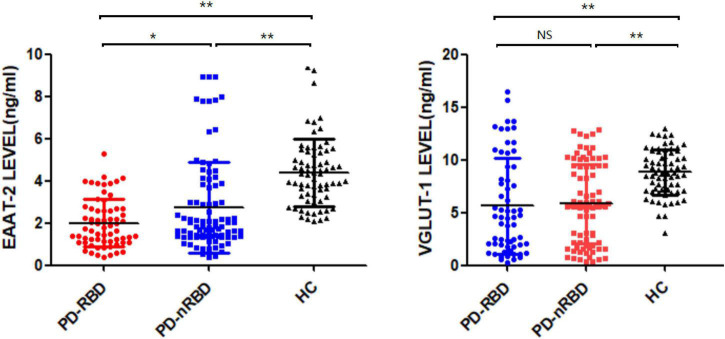
Baseline plasma levels of neuronal exosomal VGLUT-1 and EAAT-2 in PD patients with and without RBD (PD-RBD and PD-nRBD) and healthy controls (HCs). The plasma levels of neuron-derived exosomal EAAT-2 and VGLUT-1 were significant decreased in PD patients compared with HCs. The plasma levels of neuron-derived exosomal EAAT-2 was significantly lower in PD with RBD group, *P < 0.05, **P < 0.001.

We measured the plasma levels of NDE EAAT-2 and VGLUT-1 at baseline, and the plasma levels of both NDE EAAT-2 and VGLUT-1 were significantly lower in PD patients than in HCs. Between the PD-RBD and PD-nRBD groups, there was no significant difference in plasma levels of NDE VGLUT-1 (*P* = 0.713), while plasma levels of NDE EAAT-2 were significantly lower in the PD-RBD group than in the PD-nRBD group (*P* = 0.012) (shown in Figure 3).

### Cognitive decline and conversion to cognitive impairment during follow-up

**FIGURE 4:**
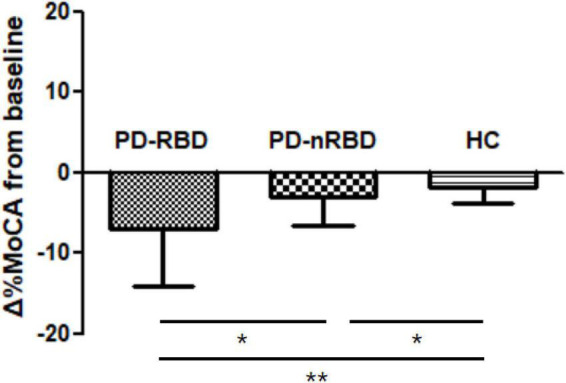
Cognitive decline by Δ%MoCA from baseline to 36 month in PD patients with and without RBD (PD-RBD and PD-nRBD) and healthy controls (HCs). *P* value was assessed using independent *t*-test between each group, *P < 0.05, **P < 0.001.

**FIGURE 5:**
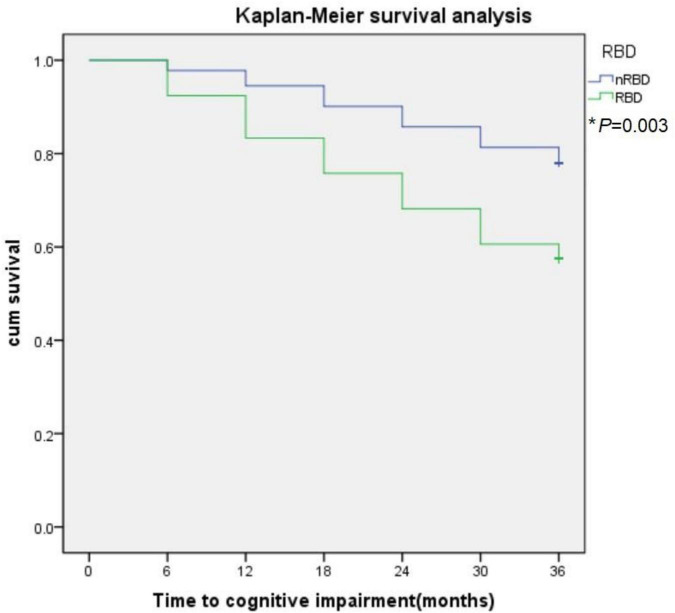
The effect of RBD on global cognitive decline in PD subjects. RBD predict conversion to cognitive impairment (CI). Kaplan-Meier survival analysis was used to estimate the effects of RBD on the progression to CI. *P value statistically significant.

During the 3-year follow-up, PD-RBD patients had a faster cognitive decline than PD-n RBD patients (*P* = 0.001) and HCs (*P* < 0.001) (Figure 4). PD-RBD patients were more likely to develop CI than PD-nRBD patients. In the Kaplan–Meier survival analysis, the time between normal cognition to the development of CI was significantly different between PD patients with and without RBD, suggesting an effect of RBD on the risk of developing CI (*P* = 0.003; Figure 5).

### Baseline plasma levels of neuron-derived exosomal excitatory amino acid transporter-2 as a predictor of cognitive decline in Parkinson’s disease patients with RBD

We further found that baseline plasma levels of NDE EAAT-2 predicted the occurrence of global cognitive decline (Δ%MoCA) over 3 years in the PD-RBD group (β = 0.064, *P* = 0.003) but not in the HC and PD-nRBD groups (shown in Table 3).

**TABLE 3:** EAAT-2 and RBD as predictors of global cognitive decline in PD patients.

	β	*P*-value
EAAT-2 (PD-RBD)	0.064 ± 0.013	0.003*
EAAT-2 (PD-nRBD)	0.001 ± 0.007	0.565
EAAT-2 (HCs)	0.024 ± 0.003	0.520

Data are shown as coefficient (β) ± (SE). In these linear effects models, Δ%MoCA is the dependent variable and plasma levels of NDE EAAT-2 is the independent variable, with age, education, disease duration and baseline UPDRS part III score as covariates. *P value statistically significant.

## Discussion

To the best of our knowledge, we are the first to investigate the relationship between plasma levels of NDE EAAT-2 and VGLUT-1 and cognitive function in PD patients with and without RBD. In our study of newly diagnosed cognitively normal PD patients, we found a significant association between RBD at baseline and the rate of cognitive decline during the 3-year follow-up. Patients with RBD had greater rates of decline in measures of global cognition. Baseline plasma levels of NDE EAAT-2 and VGLUT-1 were significant decreased in PD patients than in HCs. With further analysis, plasma levels of NDE EAAT-2 may serve as a predictor of cognitive decline in PD patients with RBD.

RBD is highly associated with cognitive decline in PD. Early studies have shown that RBD predicts CI in both early and advanced PD without dementia (Vendette et al., 2007; Chahine et al., 2016; Liu et al., 2019). In our study, on the basis of PSG, 42.04% of treatment-naïve cognitively normal PD patients were diagnosed with RBD. Along with other studies, when analyzing the effect of RBD on cognition, we confirmed that RBD was associated with global cognitive decline in PD patients. With additional analyses between the PD-RBD group and PD-nRBD group, a higher proportion of PD-RBD patients had clinically impaired performance than PD-nRBD patients on visuospatial function and memory.

The mechanisms underlying the association between CI and RBD in PD remain to be determined. The generator of REM-associated atonia is located in glutamatergic neurons of the pontine sublaterodorsal nucleus (SLD) (Luppi et al., 2011, 2013). Glutamatergic pathways also play a key role in the functional organization of neuronal circuits involved in PD. Functional interactions between the dopaminergic and glutamatergic systems in the brain regulate motor function, positive reinforcement, attention, and working memory. In PD, the degeneration of nigrostriatal dopaminergic neurons causes chain reactions that ultimately result in glutamatergic overstimulation. Relative glutamate levels in PD animal models have been shown to change within the striatum in a time-dependent manner after subacute treatment with MPTP (Pflibsen et al., 2015). In PD patients abnormal levels of glutamate has also been observed. Kashani et al. (2007) analyzed the expression of VGLUT-1 and VGLUT-2 in autopsy tissues of PD patients and matched controls, VGLUT-1 was dramatically decreased in the prefrontal and temporal cortices. However, there are still no blood-based markers of the relevant pathological mechanisms occurring in the brain that are used in clinical practice. Recently, Jiang et al. (2020) reported that serum neuronal exosomes predict and differentiate PD from atypical parkinsonism, and their findings demonstrated that protein cargoes in L1CAM-positive extracellular vesicles exhibit distinct compositions in neurodegenerative diseases that predate the clinical phase and offer a promising means to develop blood-based predictive markers of the relevant brain pathology. According to these findings, we analyzed the plasma levels of NDE EAAT-2 and VGLUT-1 in PD patients and HCs, and consistent with the early findings in mouse models and PD patients, the plasma levels of NDE EAAT-2 and VGLUT-1 were significantly lower in PD patients than in HCs. The major pathological hallmark of PD is the accumulation of insoluble α-synuclein (α-syn). Synaptic expression of α-syn was mostly accompanied by expression of VGLUT-1 (Taguchi et al., 2016). Amyloid β pathology is associated with CI in PD. It has been reported that significant decreases in the density of VGLUT-1 were found in the conus ammonis 1 region of the hippocampus in mice after Aβ1-42 injection, indicating that Aβ1-42 induces brain region- and layer-specific expression changes in glutamatergic transporters (Yeung et al., 2020). In out study, we analyzed the association between baseline plasma levels of NDE VGLUT-1 and cognitive decline in PD patients, there was no significant difference.

Specific glutamate transporters named excitatory amino acid transporters are responsible for the reuptake of glutamate, preventing non-physiological spillover from the synapse (O’Donovan et al., 2017). EAAT-2 (named GLT-1 in rodent brain), responsible for 90% of the brain glutamate reuptake, is highly expressed on astrocytes but also on neurons (Furness et al., 2008). Dysfunctional expression of glutamate transporters leads to an increase in extracellular glutamate, which causes aberrant synaptic signaling, leading to neuronal excitotoxicity and death. Glutamate excitotoxicity is a well-established pathogenesis of PD. Compelling evidence has demonstrated aberrant glutamate reuptake in animal models of PD. Acute exposure to MPTP and 6-hydroxydopamine (6-OHDA) neurotoxins is largely used to investigate biochemical and cellular dysfunctions in PD cellular and animal models (Iovino et al., 2020). In mice intraperitoneally injected with MPTP, the animal model displays typical behavioral and histopathological deficits of PD coupled with downregulation of GLT-1 protein and mRNA levels, extracellular glutamate accumulation, excitotoxicity, and astrocytic and microglial reactivity (Zhang et al., 2017). Similar to MPTP, the injection of 6-OHDA in the rat striatum causes a downregulation of GLT-1 and glutamate/aspartate transporter (GLAST) expression in the striatum (Chung et al., 2008). Inhibition of GLT-1 expression exacerbated dopamine neuron loss and motor dysfunction (Wang et al., 2018). Zhang et al. (2020) generated a novel mouse model of PD with progressive motor deficits and nigral DA neuronal death via targeted knockdown of GLT-1 in the substantia nigra, revealing the role of GLT-1 in PD pathogenesis. A recent study showed that α-syn-containing EVs released by red blood cells (RBC-EVs) from PD patients exacerbate the aggregation of α-syn in the mouse brain (Sheng et al., 2020). More interestingly, RBC-EV-induced oligomeric α-syn pathology in astrocytes affects glutamate clearance via EAAT-2, revealing the dysfunction of EAAT-2 in PD patients (Sheng et al., 2020). Along with these studies, we found that plasma levels of NDE EAAT-2 were significant decreased in PD patients, and it was even lower in PD-RBD group.

Excitatory amino acid transporter-2 also plays an essential role in cognitive functions. Hsu et al. (2015) reported a relationship between ceftriaxone-induced GLT-1 expression and improved cognition in a PD rat model. With ceftriaxone treatment, deficits in working memory in the T-maze test and in object recognition in the object recognition task were reversed, and increased GLT-1 expression was observed in the striatum and hippocampus in the animals. Similarly, upregulation of GLT-1 levels ameliorates neurodegeneration and cognitive deficits in a 6-OHDA-injected mouse model (Singh et al., 2018). Alzheimer’s disease (AD) pathology is related to Parkinson’s disease dementia (Halliday et al., 2014). Significant functional loss of EAAT-2 has been reported to correlate with the severity of CI in AD patients and animal models (Takahashi et al., 2015). Neurons of the sublaterodorsal tegmental nucleus triggering paradoxical sleep are glutamatergic (Clément et al., 2011). As previously mentioned, RBD might be due to the neurodegeneration of glutamate neurons involved in paradoxical sleep (Luppi et al., 2011, 2013; Wang et al., 2021). Since glutamate plays an important role in both RBD and cognitive decline in PD, we analyzed the plasma levels of NDE EAAT-2 in PD patients with RBD. Compared to the PD-nRBD group, the plasma levels of NDE EAAT-2 were significantly lower in the PD-RBD group. With further analysis, we found that baseline plasma levels of NDE EAAT-2 predicted global cognitive decline (Δ%MoCA) over 3 years in the PD-RBD group. These findings support our hypothesis that in PD-RBD patients, glutamatergic hyperactivity may play a role in CI.

Study limitations should be addressed. In our study, we measured the plasma levels of NDE EAAT-2, and we did not have additional information, such as pathology or CSF data, to confirm the results. Second, our sample size was relatively small, and replication and validation of the results in a larger independent sample is needed. Finally, plasma samples were not collected during the follow-up and will be included in our future study.

In summary, PD patients with RBD experience greater rates of cognitive decline than those without RBD, specifically on measures of visuospatial function and memory. Baseline plasma levels of neuron-derived exosomal EAAT-2 are associated with cognitive decline in PD patients with RBD and may serve as a predictor of cognitive decline in PD patients with RBD. These findings offer new evidence suggesting that glutamatergic hyperactivity may play a role in CI in PD patients.

## Data availability statement

The raw data supporting the conclusions of this article will be made available by the authors, without undue reservation.

## Ethics statement

The studies involving human participants were reviewed and approved by the Ethics Committee of Weihai Municipal Hospital. The patients/participants provided their written informed consent to participate in this study.

## Author contributions

JbZ and ZL designed and conceptualized the study. BL, HS, ML, JwZ, XL, and RY had a major role in the acquisition of data. TS analyzed the data. BL drafted the manuscript. JbZ revised the manuscript for intellectual content. All authors read and approved the final manuscript.
